# 5-Bromo-3-(indan-1-yl­oxy)pyridin-2-amine

**DOI:** 10.1107/S1600536811005332

**Published:** 2011-02-19

**Authors:** Sujin Cho-Schultz, John C. Kath, Curtis Moore, Arnold L. Rheingold, Alex Yanovsky

**Affiliations:** a Pfizer Global Research and Development, La Jolla Labs, 10770 Science Center Drive, San Diego, CA 92121, USA; b Department of Chemistry and Biochemistry, University of California, San Diego, 9500 Gilman Drive, La Jolla, CA 92093, USA

## Abstract

The title compound, C_14_H_13_BrN_2_O, was obtained by reaction of indan-1-yl methane­sulfonate with 2-amino-5-bromo­pyridin-3-ol in the presence of caesium carbonate. The indane ring system is approximately planar [all but one of the C atoms are coplanar within 0.03 Å, the latter atom being displaced by 0.206 (2) Å from the mean plane through the remaining atoms] and forms a dihedral angle of 58.41 (4)° with the pyridine ring. In the crystal, centrosymmetrically related mol­ecules are linked into dimers by N—H⋯N hydrogen bonds.

## Related literature

For related structures with an indane group linked to a pyridine derivative through a C—O—C bridge, see: Dinçer *et al.* (2004 ▶); Lifshits *et al.* (2008 ▶).
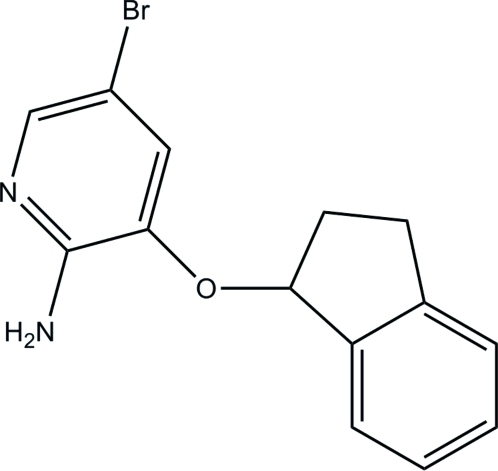


## Experimental

### 

#### Crystal data

C_14_H_13_BrN_2_O
*M* *_r_* = 305.17
Monoclinic, 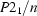

*a* = 11.3944 (18) Å
*b* = 9.4515 (15) Å
*c* = 12.438 (2) Å
β = 110.678 (2)°
*V* = 1253.2 (3) Å^3^
*Z* = 4
Mo *K*α radiation
μ = 3.27 mm^−1^
*T* = 100 K
0.21 × 0.16 × 0.08 mm

#### Data collection

Bruker APEXII CCD diffractometer
Absorption correction: multi-scan (*SADABS*; Bruker, 2001 ▶) *T*_min_ = 0.547, *T*_max_ = 0.780
23669 measured reflections
2942 independent reflections
2595 reflections with *I* > 2σ(*I*)
*R*_int_ = 0.046

#### Refinement

*R*[*F* ^2^ > 2σ(*F* ^2^)] = 0.023
*wR*(*F* ^2^) = 0.054
*S* = 1.03
2942 reflections
163 parameters
H-atom parameters constrained
Δρ_max_ = 0.38 e Å^−3^
Δρ_min_ = −0.30 e Å^−3^

### 

Data collection: *APEX2* (Bruker, 2007 ▶); cell refinement: *SAINT* (Bruker, 2007 ▶); data reduction: *SAINT*; program(s) used to solve structure: *SHELXS97* (Sheldrick, 2008 ▶); program(s) used to refine structure: *SHELXL97* (Sheldrick, 2008 ▶); molecular graphics: *SHELXTL* (Sheldrick, 2008 ▶); software used to prepare material for publication: *SHELXTL*.

## Supplementary Material

Crystal structure: contains datablocks global, I. DOI: 10.1107/S1600536811005332/rz2555sup1.cif

Structure factors: contains datablocks I. DOI: 10.1107/S1600536811005332/rz2555Isup2.hkl

Additional supplementary materials:  crystallographic information; 3D view; checkCIF report

## Figures and Tables

**Table 1:** Hydrogen-bond geometry (Å, °)

*D*—H⋯*A*	*D*—H	H⋯*A*	*D*⋯*A*	*D*—H⋯*A*
N2—H2*NA*⋯N1^i^	0.88	2.10	2.975 (2)	178

Symmetry code: (i) 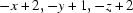.

**Fig. 1.**
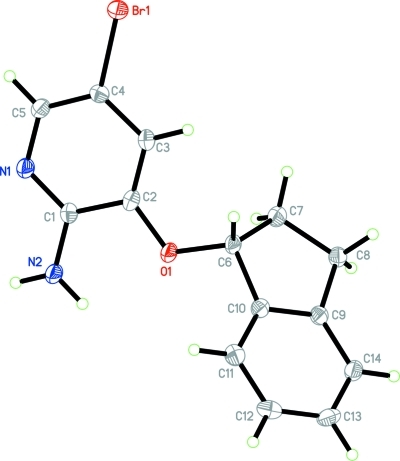
Molecular structure of the title compound, showing 50% probability displacement ellipsoids. H atoms are drawn as circles of arbitrary small radius.

**Fig. 2.**
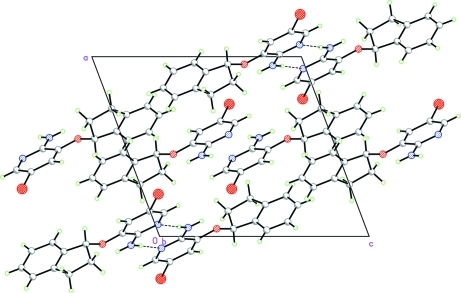
Packing diagram of the title compound viewed down the b axis. H-bonds are shown as dashed lines.

## supplementary crystallographic information

### Comment

The present study confirmed the expected structure of the title compound, the product of reaction between indan-1-yl methanesulfonate and 2-amino-5-bromopyridin-3-ol in the presence of caesium carbonate (Fig. 1).

All C atoms of the indane fragment with the exception of C7 are coplanar within 0.03 Å; the latter atom is displaced by 0.206 (2) Å from the mean plane based on all the remaining atoms of the bicyclic system. The O1 atom deviates from this plane by 1.008 (2) Å in the same direction as the C7 atom. The central C2—O1—C6 bridge is in fact coplanar with the pyridine ring so that the N1/C1/C2/C3/C3/C4/C5/O1/C6 fragment is planar within 0.01 Å and its plane forms the dihedral angle of 57.6 (3)° with the above mentioned indane plane. It is noteworthy, that general conformations of a few other structurally studied molecules featuring indane group linked to pyridine derivatives through the C—O—C bridge (Dinçer *et al.*, 2004; Lifshits *et al.*, 2008) bear close resemblance to that of the molecule of the title compound.

The N2—H2NA···N1^i^ bonds [symmetry code (i): 2 - *x*, 1 - *y*, 2 - *z*] (Table 1) link molecules in the crystal of the title compounds into centrosymmetric dimers (Fig. 2). One more intermolecular contact N2—H2NB···Br1^ii^ [symmetry code (ii): *x* - 1/2, 1.5 - *y*, *z* - 1/2] may also play certain role in the stability of the packing, although corresponding interaction seems to be too weak to be qualified as one more independent H-bond.

### Experimental

To a solution of 2-amino-5-bromopyridin-3-ol (1.640 g, 8.48 mmol) in 42 ml of DMF was added 2,3-dihydro-1*H*-inden-1-yl methanesulfonate (0.9 g, 4.24 mmol) and caesium carbonate (1.380 g, 4.24 mmol) and heated to 60°C overnight. The reaction mixture was quenched with water and the aqueous layer was extracted with EtOAc (3 *x* 20 ml). The organic layers were combined, dried over MgSO_4_, filtered and concentrated. The product was purified by flash chromatography (silica gel, 10–50% EtOAc/heptane) to give 525 mg (46%) 5-bromo-3-(2,3-dihydro-1*H*-inden-1-yloxy)pyridin-2-amine as a white solid.

The colorless crystals were grown by slow cooling of the solution of the title compound in boiling dichloroetane.

### Refinement

All H atoms were placed in geometrically calculated positions (N—H 0.88 Å, C—H 0.95 Å, 0.99 Å and 1.00 Å for aromatic, methylene and methine groups respectively) and included in the refinement in the riding motion approximation. The *U*_iso_(H) were set to 1.2*U*_eq_ of the carrying atom.

### Crystal data

**Table:**

C_14_H_13_BrN_2_O	*F*(000) = 616
*M**_r_* = 305.17	*D*_x_ = 1.617 Mg m^−^^3^
Monoclinic, *P*2_1_/*n*	Mo *K*α radiation, λ = 0.71073 Å
Hall symbol: -P 2yn	Cell parameters from 9797 reflections
*a* = 11.3944 (18) Å	θ = 2.8–27.7°
*b* = 9.4515 (15) Å	µ = 3.27 mm^−^^1^
*c* = 12.438 (2) Å	*T* = 100 K
β = 110.678 (2)°	Block, colorless
*V* = 1253.2 (3) Å^3^	0.21 × 0.16 × 0.08 mm
*Z* = 4	

### Data collection

**Table:**

Bruker APEXII CCD diffractometer	2942 independent reflections
Radiation source: fine-focus sealed tube	2595 reflections with *I* > 2σ(*I*)
graphite	*R*_int_ = 0.046
φ and ω scans	θ_max_ = 28.3°, θ_min_ = 2.1°
Absorption correction: multi-scan (*SADABS*; Bruker, 2001)	*h* = −15→14
*T*_min_ = 0.547, *T*_max_ = 0.780	*k* = −12→12
23669 measured reflections	*l* = −16→15

### Refinement

**Table:**

Refinement on *F*^2^	Primary atom site location: structure-invariant direct methods
Least-squares matrix: full	Secondary atom site location: difference Fourier map
*R*[*F*^2^ > 2σ(*F*^2^)] = 0.023	Hydrogen site location: inferred from neighbouring sites
*wR*(*F*^2^) = 0.054	H-atom parameters constrained
*S* = 1.03	*w* = 1/[σ^2^(*F*_o_^2^) + (0.0191*P*)^2^ + 0.7815*P*] where *P* = (*F*_o_^2^ + 2*F*_c_^2^)/3
2942 reflections	(Δ/σ)_max_ = 0.001
163 parameters	Δρ_max_ = 0.38 e Å^−^^3^
0 restraints	Δρ_min_ = −0.30 e Å^−^^3^

### Special details

**Table:**

Geometry. All s.u.'s (except the s.u. in the dihedral angle between two l.s. planes) are estimated using the full covariance matrix. The cell s.u.'s are taken into account individually in the estimation of s.u.'s in distances, angles and torsion angles; correlations between s.u.'s in cell parameters are only used when they are defined by crystal symmetry. An approximate (isotropic) treatment of cell s.u.'s is used for estimating s.u.'s involving l.s. planes.
Refinement. Refinement of *F*^2^ against ALL reflections. The weighted *R*-factor *wR* and goodness of fit *S* are based on *F*^2^, conventional *R*-factors *R* are based on *F*, with *F* set to zero for negative *F*^2^. The threshold expression of *F*^2^ > 2σ(*F*^2^) is used only for calculating *R*-factors(gt) *etc*. and is not relevant to the choice of reflections for refinement. *R*-factors based on *F*^2^ are statistically about twice as large as those based on *F*, and *R*- factors based on ALL data will be even larger.

### Fractional atomic coordinates and isotropic or equivalent isotropic displacement parameters (Å^2^)

**Table:**

	*x*	*y*	*z*	*U*_iso_*/*U*_eq_	
Br1	1.235622 (16)	1.080477 (17)	1.070416 (15)	0.02063 (6)	
O1	0.95752 (11)	0.77404 (12)	0.71352 (10)	0.0192 (3)	
N1	1.06647 (13)	0.68706 (14)	1.01301 (12)	0.0179 (3)	
N2	0.95112 (15)	0.56292 (15)	0.84811 (13)	0.0227 (3)	
H2NA	0.9477	0.4894	0.8903	0.027*	
H2NB	0.9150	0.5591	0.7729	0.027*	
C1	1.01219 (16)	0.68247 (17)	0.89913 (14)	0.0163 (3)	
C2	1.01793 (15)	0.79884 (17)	0.82822 (14)	0.0165 (3)	
C3	1.08206 (16)	0.91835 (17)	0.87805 (15)	0.0171 (3)	
H3	1.0868	0.9979	0.8331	0.020*	
C4	1.14048 (16)	0.91900 (16)	0.99797 (15)	0.0161 (3)	
C5	1.13089 (16)	0.80499 (17)	1.06228 (15)	0.0180 (3)	
H5	1.1704	0.8086	1.1435	0.022*	
C6	0.95743 (16)	0.88611 (17)	0.63483 (14)	0.0168 (3)	
H6	1.0431	0.9283	0.6559	0.020*	
C7	0.85977 (17)	1.00246 (18)	0.62819 (15)	0.0209 (4)	
H7A	0.8003	0.9696	0.6647	0.025*	
H7B	0.9019	1.0892	0.6681	0.025*	
C8	0.78992 (16)	1.03247 (17)	0.49914 (15)	0.0184 (3)	
H8A	0.6983	1.0385	0.4815	0.022*	
H8B	0.8193	1.1220	0.4759	0.022*	
C9	0.82218 (16)	0.90756 (16)	0.43914 (15)	0.0164 (3)	
C10	0.91601 (15)	0.82524 (17)	0.51553 (14)	0.0163 (3)	
C11	0.96492 (16)	0.70772 (17)	0.47841 (15)	0.0200 (4)	
H11	1.0284	0.6517	0.5316	0.024*	
C12	0.91930 (17)	0.67388 (18)	0.36230 (16)	0.0225 (4)	
H12	0.9523	0.5947	0.3353	0.027*	
C13	0.82497 (17)	0.75614 (19)	0.28514 (15)	0.0224 (4)	
H13	0.7942	0.7322	0.2059	0.027*	
C14	0.77560 (17)	0.87233 (18)	0.32274 (15)	0.0204 (4)	
H14	0.7109	0.9272	0.2699	0.025*	

### Atomic displacement parameters (Å^2^)

**Table:**

	*U*^11^	*U*^22^	*U*^33^	*U*^12^	*U*^13^	*U*^23^
Br1	0.02147 (10)	0.01833 (9)	0.01970 (10)	−0.00397 (7)	0.00432 (7)	−0.00082 (6)
O1	0.0247 (7)	0.0182 (6)	0.0125 (6)	−0.0039 (5)	0.0038 (5)	0.0033 (4)
N1	0.0195 (7)	0.0179 (7)	0.0156 (7)	−0.0011 (6)	0.0055 (6)	0.0024 (5)
N2	0.0298 (9)	0.0198 (7)	0.0140 (7)	−0.0074 (6)	0.0021 (6)	0.0035 (6)
C1	0.0150 (8)	0.0174 (7)	0.0168 (8)	0.0007 (6)	0.0058 (7)	0.0029 (6)
C2	0.0149 (8)	0.0209 (8)	0.0137 (8)	0.0019 (6)	0.0052 (6)	0.0030 (6)
C3	0.0178 (8)	0.0168 (8)	0.0172 (9)	0.0013 (6)	0.0070 (7)	0.0039 (6)
C4	0.0141 (8)	0.0155 (7)	0.0184 (8)	−0.0002 (6)	0.0053 (7)	−0.0013 (6)
C5	0.0187 (9)	0.0201 (8)	0.0145 (8)	0.0009 (7)	0.0049 (7)	0.0003 (6)
C6	0.0195 (9)	0.0169 (7)	0.0140 (8)	−0.0022 (6)	0.0057 (7)	0.0033 (6)
C7	0.0257 (10)	0.0202 (8)	0.0161 (9)	0.0025 (7)	0.0067 (7)	0.0009 (7)
C8	0.0184 (9)	0.0171 (8)	0.0180 (9)	0.0002 (6)	0.0045 (7)	0.0020 (6)
C9	0.0170 (8)	0.0158 (7)	0.0166 (8)	−0.0037 (6)	0.0062 (7)	0.0018 (6)
C10	0.0159 (8)	0.0177 (8)	0.0158 (8)	−0.0032 (6)	0.0061 (7)	0.0012 (6)
C11	0.0179 (9)	0.0193 (8)	0.0225 (9)	−0.0002 (7)	0.0067 (7)	0.0013 (7)
C12	0.0233 (9)	0.0200 (8)	0.0262 (10)	−0.0048 (7)	0.0113 (8)	−0.0068 (7)
C13	0.0256 (10)	0.0258 (9)	0.0154 (9)	−0.0086 (7)	0.0066 (7)	−0.0050 (7)
C14	0.0213 (9)	0.0199 (8)	0.0169 (9)	−0.0040 (7)	0.0028 (7)	0.0029 (7)

### Geometric parameters (Å, °)

**Table:**

Br1—C4	1.9044 (16)	C7—C8	1.545 (2)
O1—C2	1.368 (2)	C7—H7A	0.9900
O1—C6	1.4419 (19)	C7—H7B	0.9900
N1—C1	1.331 (2)	C8—C9	1.510 (2)
N1—C5	1.356 (2)	C8—H8A	0.9900
N2—C1	1.361 (2)	C8—H8B	0.9900
N2—H2NA	0.8800	C9—C10	1.391 (2)
N2—H2NB	0.8800	C9—C14	1.395 (2)
C1—C2	1.426 (2)	C10—C11	1.393 (2)
C2—C3	1.369 (2)	C11—C12	1.388 (3)
C3—C4	1.402 (2)	C11—H11	0.9500
C3—H3	0.9500	C12—C13	1.397 (3)
C4—C5	1.369 (2)	C12—H12	0.9500
C5—H5	0.9500	C13—C14	1.388 (3)
C6—C10	1.504 (2)	C13—H13	0.9500
C6—C7	1.546 (2)	C14—H14	0.9500
C6—H6	1.0000		
			
C2—O1—C6	117.49 (13)	C6—C7—H7A	110.4
C1—N1—C5	118.79 (14)	C8—C7—H7B	110.4
C1—N2—H2NA	120.0	C6—C7—H7B	110.4
C1—N2—H2NB	120.0	H7A—C7—H7B	108.6
H2NA—N2—H2NB	120.0	C9—C8—C7	104.11 (13)
N1—C1—N2	119.49 (15)	C9—C8—H8A	110.9
N1—C1—C2	121.84 (15)	C7—C8—H8A	110.9
N2—C1—C2	118.66 (15)	C9—C8—H8B	110.9
O1—C2—C3	127.25 (15)	C7—C8—H8B	110.9
O1—C2—C1	113.39 (14)	H8A—C8—H8B	109.0
C3—C2—C1	119.35 (15)	C10—C9—C14	119.63 (16)
C2—C3—C4	117.53 (15)	C10—C9—C8	111.25 (15)
C2—C3—H3	121.2	C14—C9—C8	129.04 (16)
C4—C3—H3	121.2	C9—C10—C11	121.40 (16)
C5—C4—C3	120.80 (15)	C9—C10—C6	110.97 (14)
C5—C4—Br1	120.25 (13)	C11—C10—C6	127.55 (15)
C3—C4—Br1	118.94 (12)	C12—C11—C10	118.81 (16)
N1—C5—C4	121.67 (16)	C12—C11—H11	120.6
N1—C5—H5	119.2	C10—C11—H11	120.6
C4—C5—H5	119.2	C11—C12—C13	120.06 (16)
O1—C6—C10	108.27 (13)	C11—C12—H12	120.0
O1—C6—C7	112.76 (14)	C13—C12—H12	120.0
C10—C6—C7	104.49 (14)	C14—C13—C12	120.92 (16)
O1—C6—H6	110.4	C14—C13—H13	119.5
C10—C6—H6	110.4	C12—C13—H13	119.5
C7—C6—H6	110.4	C13—C14—C9	119.18 (16)
C8—C7—C6	106.45 (14)	C13—C14—H14	120.4
C8—C7—H7A	110.4	C9—C14—H14	120.4

### Hydrogen-bond geometry (Å, °)

**Table:**

*D*—H···*A*	*D*—H	H···*A*	*D*···*A*	*D*—H···*A*
N2—H2NA···N1^i^	0.88	2.10	2.975 (2)	178

Symmetry codes: (i) −*x*+2, −*y*+1, −*z*+2.
